# Ultranarrow Graphene Nanoribbons toward Oxygen Reduction and Evolution Reactions

**DOI:** 10.1002/advs.201801375

**Published:** 2018-11-06

**Authors:** Jian Zhang, Yuanmiao Sun, Jiawei Zhu, Zhonghui Gao, Shuzhou Li, Shichun Mu, Yunhui Huang

**Affiliations:** ^1^ State Key Laboratory of Material Processing and Die & Mould Technology School of Materials Science and Engineering Huazhong University of Science and Technology Wuhan 430074 P. R. China; ^2^ School of Materials Science and Engineering Nanyang Technological University Singapore 639798 Singapore; ^3^ State Key Laboratory of Advanced Technology for Materials Synthesis and Processing Wuhan University of Technology Wuhan 430070 P. R. China

**Keywords:** graphene, graphene nanoribbons, oxygen evolution reaction, oxygen reduction reaction

## Abstract

Identification of catalytic sites for oxygen reduction and evolution reactions (ORR/OER) is critical to rationally develop highly efficient bifunctional carbon‐based metal‐free electrocatalyst. Here, a unique defect‐rich N‐doped ultranarrow graphene nanoribbon with a high aspect ratio that exhibits excellent ORR/OER bifunctional activities and impressive long‐term cycling stability in Zn–air batteries is successfully fabricated. Density functional theory calculations indicates that the topological defects (e.g., pentagons and heptagons) cooperated with pyridinic‐N dopants on the edges are more favorable to electrocatalytic activity toward ORR and OER. This work provides a new design principle for carbon‐based electrocatalytic nanomaterials.

The fast consumption of traditional fuels and the environmental problems have urgently required the development of new and sustainable energy technologies, such as regenerative fuel cells and rechargeable metal–air batteries.^1^, ^2^ In these devices, the sluggish kinetics of oxygen reduction and evolution reactions (ORR/OER) on the cathode severely reduce the overall efficiency.^3^, ^4^ Therefore, catalysts are required for both ORR and OER. Generally, Pt‐based catalysts are most efficient for ORR, while Ir/Ru‐based catalysts are highly active toward OER.^1^, ^5^ However, these precious metals have several disadvantages such as high cost, limited resource and inferior durability, which hamper their widespread and large‐scale applications. Thus, considerable efforts have been explored to search for non‐precious metal or even metal‐free catalysts for ORR and OER.

Along with the extensive research efforts, heteroatom‐doped (N, O, B, P, S, F, etc.) carbon nanomaterials, such as carbon nanospheres and nanotubes, graphene nanosheets and nanoribbons, have attracted enormous interest as promising alternatives of precious metal catalysts due to their economic viability, tunable structure, facile preparation, chemical stability, and multifunctionality.^1^, ^6^, ^7^, ^8^, ^9^ The electrocatalytic activities of heteroatom‐doped carbon nanomaterials are supposed to be ascribed to the electroneutrality break and spin distribution of the sp^2^ carbon plane induced by the heteroatom doping.^1^, ^8^, ^10^, ^11^ Recently, it has been stated that the intrinsic edges and defects (zigzag and armchair edge, pentagonal, heptagonal, etc.) in carbon nanomaterials show considerable electrocatalytic activities.^8^, ^12^, ^13^, ^14^ As reported, even dopant‐free carbon nanomaterials can deliver good ORR or/and OER activities.^14^, ^15^ The carbon nanomaterials with higher edge heteroatom doping and richer defects are likely to be more active.^2^, ^8^, ^16^, ^17^ However, the exact role of the defects in the N‐doped carbon nanomaterials and the underlying mechanism of ORR and OER are still unclear and controversial. At this point, exploring the origin and role of nanocarbon active sites are fundamentally important for accelerating the development and rational design of advanced carbon‐based electrocatalysts.

In this work, we developed defect‐rich N‐doped ultranarrow graphene nanoribbons (DN‐UGNR) by chemical oxidation and unzipping of carbon nanotubes (CNT),^18^, ^19^ followed by ammonia injection at high temperature, as detailedly illustrated in Scheme S1 of the Supporting Information. Owing to the high aspect ratio of 1D nanostructure, abundant edges/defects and pyridinic‐N dopants, DN‐UGNR exhibits excellent ORR and OER activities in alkaline condition. First‐principles calculations were carried out to explain the observed outstanding electrocatalytic activities. We further employed DN‐UGNR as a bifunctional catalyst to prepare Zn–air batteries and obtained a better discharge performance and more stable long‐term cyclability compared to the electrode made from the mixed Pt/C and RuO_2_ catalyst.

The morphology of DN‐UGNR was investigated by transmission electron microscope (TEM). **Figure** **1**a presents a single nanoribbon that has a high length–width ratio. No clear walls or cavities are observed in contrast to the typical CNT (Figure S1, Supporting Information), indicating that the original CNT has been fully unzipped by the violent reactions.^18^, ^20^ Estimated from the TEM images (Figure 1b; Figure S2, Supporting Information), the widths of these nanoribbons mainly range from 1 to 5 nm, which are narrower than most of the reported carbon nanoribbons (Figure 1e). Such high aspect ratio of DN‐UGNR indicates that it has plenty of exposed edge atoms and defects, which is favorable to increase the catalytic active density.^8^, ^16^ Additionally, these nanoribbons are self‐assembled into a porous reticular architecture (Figure 1b–d), which facilitates the mass and electron transfer for the electrocatalysis.^6^, ^13^, ^21^ Such excellent porous structure of DN‐UGNR can also be verified by the N_2_ adsorption and desorption isotherm measurements (specific surface area: 679 m^2^ g^−1^) and pore size distribution (Figure 1f).

**Figure 1:**
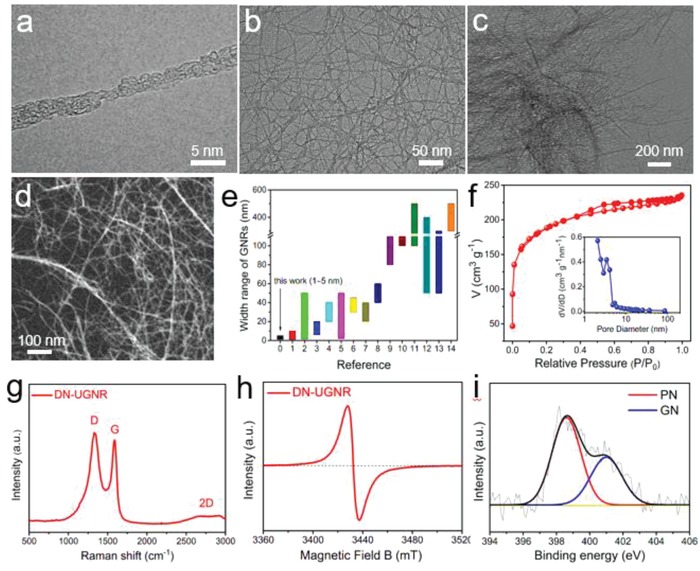
a–d) TEM image of DN‐UGNR. e) The width range of DN‐UGNR and other literature reported GNRs, as derived from Table S1 of the Supporting Information. f) Nitrogen adsorption–desorption isotherms of DN‐UGNR, the inset is pore size distribution. g) Raman, h) EPR, and the fitted N 1s spectra of DN‐UGNR.

Figure 1g shows the Raman spectrum of DN‐UGNR. Compared to pristine CNT (Figure S3, Supporting Information), DN‐UGNR displays a broad D‐band and high‐intensity ratio of the D to G peak (*I*_D_/*I*_G_ = 1.08), suggesting low graphitization degree and abundant defective sites in DN‐UGNR,^14^, ^15^ which can be also confirmed by the low intensity of the (002) peak in X‐ray diffraction pattern (XRD, Figure S4, Supporting Information).^9^ Besides, the electron paramagnetic resonance (EPR) measurement of DN‐UGNR (Figure 1h) presents a well‐defined symmetric peak, further demonstrating the defective feature.^22^ Figure S5 (Supporting Information) shows X‐ray photoelectron spectroscopy (XPS) of DN‐UGNR. The N 1s is fitted into two peaks corresponding to pyridinic‐N (PN, 62.7 at%) and graphitic‐N (GN, 32.3 at%) (Figure 1i; Table S2, Supporting Information). Generally, the high content of PN in carbon matrix is desirable for high electrocatalytic activity.^13^, ^17^, ^22^, ^23^

The ORR activity of the catalyst was explored by measuring the linear sweep voltammetry (LSV) on rotating disk electrode (RDE) in an O_2_‐saturated 0.1 m KOH aqueous electrolyte. For comparison, the pristine CNT, N‐doped CNT (N‐CNT), undoped defective UGNR (D‐UGNR), and commercial Pt/C (20%) were also tested. As displayed in **Figure** **2**a, the ORR activity of CNT is very poor. Even with N doping (N‐CNT), the ORR activity only slightly increases. This may be attributed to its lack of defective sites (Figure S6, Supporting Information) and low active N dopants (Figure S7, Supporting Information). The DN‐UGNR catalyst displays the best ORR activity in terms of the onset and half‐wave potential (*E*_0_ = 0.957 V, *E*_1/2_ = 0.808 V). Remarkably, the *E*_0_ of DN‐UGNR is even 13 mV more positive than Pt/C catalyst, which is also higher than most of the non‐noble metal or metal‐free carbon‐based catalysts (Table S3, Supporting Information). In addition, DN‐UGNR catalyst also shows considerable ORR activity (*E*_0_ = 0.81 V) in acidic media (Figure S8, Supporting Information). Interestingly, even without N doping, D‐UGNR has a considerable ORR activity (*E*_0_ = 0.899 V, *E*_1/2_ = 0.753 V). As seen in Figure S9 (Supporting Information), the D‐UGNR catalyst possesses a similar defective feature as DN‐UGNR: broad XRD (002) peak, high *I*_D_/*I*_G_ value (1.06), and a pair of EPR symmetric peaks. Hence, it is rational to conclude that the defective sites can give rise to a well‐defined electrocatalytic activity, which agrees well with the previous works.^8^, ^13^, ^14^, ^15^, ^24^

**Figure 2:**
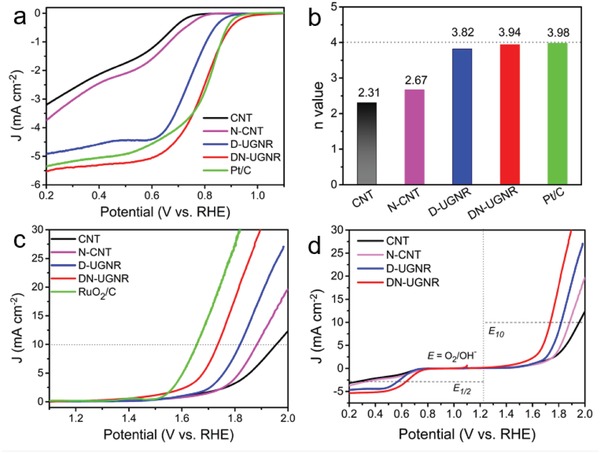
a) ORR polarization curves and b) the average electron transfer number (*n*) of CNT, N‐CNT, D‐UGNR, DN‐UGNR, and Pt/C catalysts. c) OER polarization curves for CNT, N‐CNT, D‐UGNR, DN‐UGNR, and RuO_2_/C catalysts. d) The overall polarization curves of CNT, N‐CNT, D‐UGNR, and DN‐UGNR catalysts in the whole ORR and OER region.

Further, we measured their LSV curves at different rotation rates (Figure S10a–e, Supporting Information). According to Equations (S1) and (S2) (Supporting Information),^10^, ^13^ five corresponding Koutecky–Levich (K–L) plots from 0.2 to 0.6 V are generated (Figure S10f–j, Supporting Information). From the slopes of these K–L plots, the average electron transfer number (*n*) of CNT, N‐CNT, UGNR, DN‐UGNR, and Pt/C catalyst are 2.31, 2.67, 3.82, 3.94, and 3.96, respectively. We also carried out the rotating RDE measurement for DN‐UGNR and Pt/C catalysts, as shown in Figure S11 (Supporting Information). Based on Equations (S3) and (S4) (Supporting Information),^3^, ^13^ both *n* values and H_2_O_2_ yields for DN‐UGNR are very close to those of Pt/C catalyst (Figure S12, Supporting Information). This further supports that the DN‐UGNR mainly involves in a four‐electron transfer process with high efficiency on ORR. To gain insight into the ORR kinetics of these catalysts, Tafel plots were performed (Figure S13, Supporting Information). DN‐UGNR catalyst describes the lowest Tafel slope (58 mV per decade), suggesting fast transfer of the first electron during the ORR.^10^ In addition, the durability is also important for electrocatalyst. As described in Figure S14 (Supporting Information), we performed the LSV measurements for D‐UGNR, DN‐UGNR, and Pt/C catalysts before and after the accelerated durability test of 3000 cycles. The decay of half‐wave potential of D‐UGNR and DN‐UGNR is 5 and 6 mV, respectively, much lower than that of Pt/C (21 mV), indicating that the intrinsic carbon‐based electrocatalysts have better ORR stability than noble metal Pt/C catalyst.

The OER activities of these catalysts were also checked. As reference, the noble metal ruthenium oxide mixed with carbon black (XC‐72, 20 wt%) (RuO_2_/C) was also tested. As seen in Figure 2c, the pristine CNT and N‐CNT demonstrate the poorest OER activity, represented by the largest overpotential at the geometric current density of 10 mA cm^−2^ (*E*_10_). For D‐UGNR, the OER activity (*E*_10_ = 0.595 V) is superior to those of CNT and N‐CNT catalysts, suggesting that the defects in carbon matrix also contribute to the active sites for OER.^14^, ^15^ With combination of defects and N dopants, DN‐UGNR shows an even better OER activity with *E*_10_ of 0.512 V, which is close to the RuO_2_/C and other carbon‐based electrocatalysts (Table S3, Supporting Information). Tafel plot of DN‐UGNR also shows that its OER kinetics approaches to that of RuO_2_/C catalyst (Figure S15, Supporting Information). Moreover, both D‐UGNR and DN‐UGNR reveal good OER durability (Figure S16, Supporting Information). Further, the overall electrocatalytic activity is probed by the potential gap (Δ*E*) between the potential at 10 mA cm^−2^ for OER and the *E*_1/2_ for ORR.^13^, ^17^ As summarized in Figure 2d, the DN‐UGNR shows the lowest Δ*E* value of 0.934 V compared to CNT (1.413 V), N‐CNT (1.293 V), and D‐UGNR (1.072 V), indicative of an excellent bifunctional electrocatalysis.

Furthermore, the samples with different ammonia‐treated temperatures (Supporting Information) were also investigated. As exhibited in Figure S17 (Supporting Information), DN‐UGNR (as named as DN‐UGNR‐A900) displays the highest ORR and OER activities compared to the samples with ammonia‐treated temperature at 800 °C (DN‐UGNR‐A800) and 1000 °C (DN‐UGNR‐A1000). The XPS results (Figure S18 and Table S2, Supporting Information) show that the DN‐UGNR‐A800 has the highest N content (5.5 at%), but its content of inactive oxygen atoms is very high (14.2 at%), which may reduce the conductivity and hence compromise the ORR activity.^13^, ^25^ When ammonia injection temperature increases to 1000 °C, the C—N bond may dramatically break, leading to the loss of active sites (the N content in DN‐UGNR‐A1000 is only 2.9 at%). In addition, the excessively high temperature would result in the drop of the electroactive defect sites (see Raman results in Figure S19, Supporting Information).^1^, ^26^ Thus, the optimal ammonia injection temperature is 900 °C in our case.

Such high electrocatalytic activities of DN‐UGNR in ORR and OER may result from the unique high aspect ratio of the 1D nanostructure. To confirm this, various widths of defective and N‐doped GNRs (**Figure** **3**a–d) were obtained by unzipping different diameter of carbon nanotubes followed by ammonia treatment at the same conditions. As shown in Figure 3e, the *I*_D_/*I*_G_ ratio gradually decreases with increasing width of GNR, indicating that the defects gradually decrease. Figure 3f–h shows that the ORR/OER activities and Δ*E* values gradually decrease with increasing the GNR width. These results demonstrate that the narrow width of GNR is more preferred for ORR and OER. According to Dai's research,^12^, ^14^, ^27^ the carbon atoms with higher charge and spin densities are more likely to serve as catalytically active sites, which is confirmed the high electrocatalytic property of DN‐UGNR catalyst.

**Figure 3:**
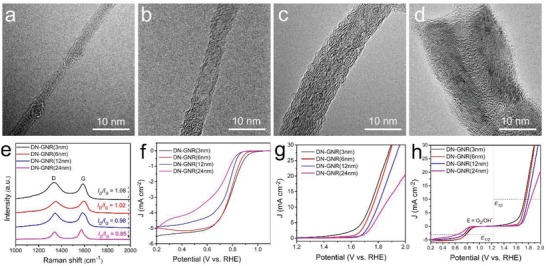
a–d) TEM images and e) Raman spectra of different width of DN‐GNR range from 3 to 24 nm. f) ORR and g) OER polarization curves, and h) the overall polarization curves of different width of DN‐GNRs.

Furthermore, considering abundant topological defects (e.g., pentagons and heptagons, as derived from carbon nanotubes)^28^ and N dopants coexist in the DN‐UGNR catalyst, which may synergistically modify the electronic properties of the surrounding carbon atoms, and provide strong binding affinity for the oxygen reaction species.^8^, ^12^, ^15^, ^27^ Herein, several possible composites or individual active sites (**Figure** **4**a; Figure S20, Supporting Information) are proposed based on first‐principles calculations.^1^, ^16^, ^24^ Typical volcano plots are constructed for both ORR and OER (Figure 4b), correlating the overpotential and the descriptor (adsorption free energy of *OH). As shown in Figure 4b, the adsorption of *OH on the pristine and the doped GNRs is too weak. When combining these defects with N dopants, they can synergistically regulate the *OH free energy toward an optimal value, rendering superb electrocatalytic activities (Z‐PN+C5 site for ORR, A‐PN+C5 site for OER) at the peak of the volcano plots. The reaction pathways of ORR on the active PN6+EC5 site and OER on the A‐PN+C5 site were predicted, as illustrated in Figure 4c,d, respectively. For ORR, the desorption of *OH tends to be the rate‐determining step (RDS). For OER, the RDS is calculated to be the transformation of *OH to *O. In order to further reveal the distinct electrocatalytic activity of ORR on Z‐PN+C5 site and OER on A‐PN+C5 site, their electronic charge density difference before and after the adsorption of the relevant species are presented (Figure 4e,f). It is found that all the intermediate species are stable on the adjacent pentagon carbon rings with PN dopants. The distortion of the pentagon carbon ring adjacent to the PN dopant with high electronegativity will result in charge redistribution and spatial curvatures, which are expected to form a permanent dipole moment.^16^ Such dipole moment offers strong binding affinity for key reaction intermediates and thus enhances the electrochemical properties. Based on the above calculations, pentagon defects with PN incorporated are expected to acquire the most efficient electrocatalytic activity toward ORR and OER.

**Figure 4:**
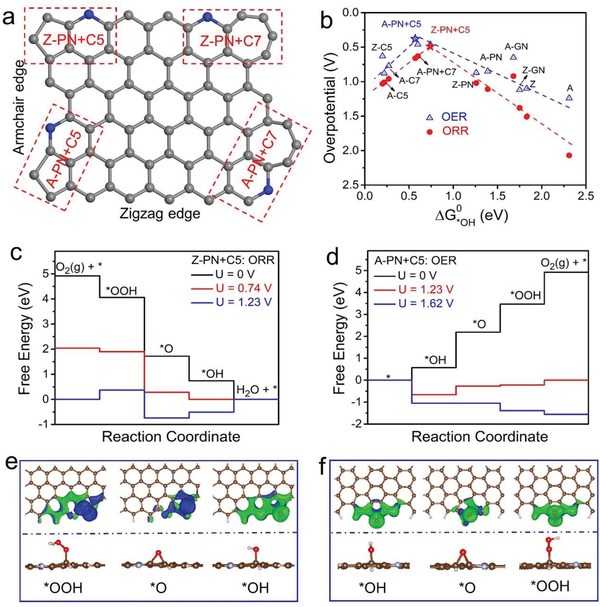
a) A schematic graphene nanoribbon with four typical composite active sites. A: armchair edge, Z: zigzag edge, PN: pyridinic‐N, C5: pentagon carbon ring, C7: heptagon carbon ring. For example, the composite of pyridinic‐N and pentagon defect on the armchair edge is labeled as A‐PN+C5. b) ORR and OER volcano plots of overpotential versus adsorption energy of *OH (Δ*G* ^0^_*OH_). c) Calculated Gibbs free energy diagrams of ORR and e) the optimized adsorption configurations of ORR intermediates (*OOH, *O, and *OH) on Z‐PN+C5 site. d) Calculated Gibbs free energy diagrams of OER and f) the optimized adsorption configurations of OER intermediates (*OOH, *O, and *OH) on A‐PN+C5 site.

As a proof of concept, the home‐made Zn–air batteries were fabricated with DN‐UGNR catalyst loaded on carbon paper as air cathode. As exhibited in **Figure** **5**a,b, the open‐circuit voltage and discharge plateaus at the discharge current density of 5 mA cm^−2^ are 1.48 and 1.31 V, respectively, superior to those of Pt/C electrode (1.43 V; and 1.28 V). In Figure 5c, the maximum power density of the battery reaches 151 mW cm^−2^, also higher than that of Pt/C electrode (126 mW cm^−2^). We further compare the performances of the Zn–air batteries based on DN‐UGNR and mixed Pt/C+RuO_2_ (1:1 by weight) catalysts. As shown in Figure 5d, during the initial cycling, the mixed noble metal electrode exhibits good discharge/charge performance, but it is not stable. With continuous cycling, the performance of the DN‐UGNR electrode becomes better than the mixed electrode and remains stable for a long time. Even after 300 cycles, the voltage gap between charge and discharge keeps almost the same as the initial cycle, suggesting the robustness of the DN‐UGNR catalyst for ORR and OER. Meanwhile, the DN‐UGNR battery also shows an excellent rate capability at various current densities from 5 to 50 mA cm^−2^ (Figure 5e). As an illustration, 22 parallel high‐power red‐light‐emitting diode (LED) lamp beads can be well powered by two series‐connected Zn–air batteries with DN‐UGNR electrode (Figure 5f). Remarkably, they can also power up a high‐voltage LED lamp bead (orange, green, and blue, Figure 5g–i), and keep a high brightness for a long duration.

**Figure 5:**
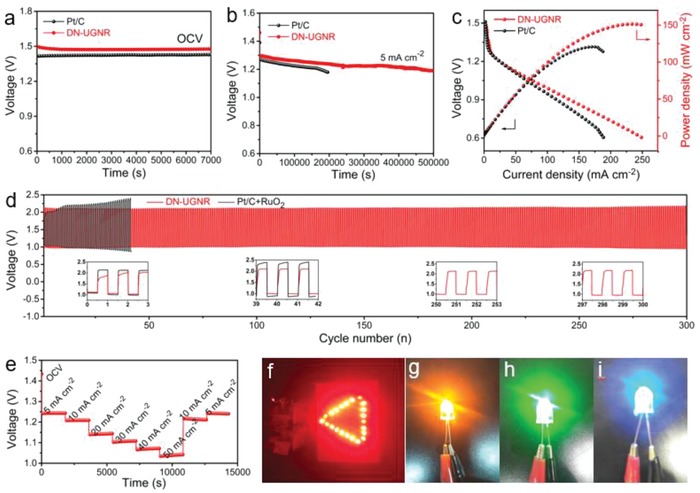
a) Open‐circuit voltage measurement, b) discharge curves, and c) polarization curve and power density plot of Zn–air batteries used DN‐UGNR and Pt/C electrodes. d) Discharge and charge cycling of rechargeable Zn–air batteries based on DN‐UGNR and the mixture of Pt/C+RuO_2_ electrodes at the current density of 5 mA cm^−2^. e) Rate capability behavior of Zn–air battery with DN‐UGNR electrode. Photograph of f) 22 parallel red and g–i) high‐voltage (orange, green, and blue, 3.0–3.2 V) LED lamp beads driven by two Zn–air batteries with the DN‐UGNR electrode connected in series.

In this study, we have successfully constructed a unique defect‐rich and pyridinic‐N dominated ultranarrow graphene nanoribbons (DN‐UGNR) toward efficient oxygen electrocatalysis. When employed DN‐UGNR as a bifunctional catalyst in the air electrode, the assembled Zn–air battery delivers a power density as high as 151 mW cm^−2^, and an outstanding discharge/charge stability with at least 300 cycles, which is much better than the mixed Pt/C+RuO_2_ electrode. Experimental and density functional theory calculations demonstrate that the excellent electrocatalytic activities of DN‐UGNR originate from the unique high aspect ratio of the 1D nanostructure, and the synergistic topological defects and PN dopants. This synergistic mechanism of defects cooperated with heteroatom dopants can provide a new avenue for the design of high‐performance carbon‐based electrocatalysts for energy conversion and storage applications.

## Experimental Section

The details of material synthesis and characterizations, device fabrications, and measurements are provided in the Supporting Information.

## Conflict of Interest

The authors declare no conflict of interest.

## Supporting information

Supplementary
