# Supplementary material for: Ultranarrow Graphene Nanoribbons toward Oxygen Reduction and Evolution Reactions
Source: Adv Sci (Weinh). 2018 Nov 6;5(12):1801375. doi: 10.1002/advs.201801375 (PMC6299708; doi:10.1002/advs.201801375)
Supplement: Supplementary file 1 — Supplementary [file ADVS-5-1801375-s001.pdf]

Copyright WILEY-VCH Verlag GmbH & Co. KGaA, 69469 Weinheim, Germany, 2018.

ADVANCED SCIENCE Open Access

Supporting Information

for *Adv. Sci.,* DOI: 10.1002/advs.201801375

Ultranarrow Graphene Nanoribbons toward Oxygen Reduction and Evolution Reactions

Jian Zhang, Yuanmiao Sun, Jiawei Zhu, Zhonghui Gao, Shuzhou Li, Shichun Mu, and Yunhui Huang**

Supporting Information

Ultranarrow Graphene Nanoribbons toward Oxygen Reduction and Evolution Reactions

Jian Zhang, Yuanmiao Sun, Jiawei Zhu, Zhonghui Gao, Shuzhou Li, Shichun Mu* and Yunhui Huang*

Dr. J. Zhang, Dr. Z, Gao and Prof. Y. Huang
State Key Laboratory of Material Processing and Die & Mould Technology, School of Materials Science and Engineering, Huazhong University of Science and Technology, Wuhan 430074, People's Republic of China
E-mail: huangyh@hust.edu.cn
Dr. J. Zhang, Dr. Y. Sun and Prof. S. Li
School of Materials Science and Engineering, Nanyang Technological University, Singapore 639798, Singapore
J. Zhu and Prof. S. Mu
State Key Laboratory of Advanced Technology for Materials Synthesis and Processing, Wuhan University of Technology, Wuhan 430070, People's Republic of China
E-mail: msc@whut.edu.cn

Experimental Section

Firstly, 0.2 g of the commercial single-walled carbon nanotubes (CNTs, length: ca. 15 μm, diameter: 1-3 nm, Shenzhen Nanotech Port Co., Ltd.) were put into the mixture solution of concentrated H_2SO_4 (50 mL) and H_3PO_4 (7 mL) for two hours. And then, 1.0 g of $KMnO_4$ was very slowly (about two hours) added to the above solution with continuous stirring (500 rpm). Followed, the above-mixed solution was kept into the oil bath at 70 °C for 1 hour with continuous stirring. After cooled down to room temperature, the mixture was poured into the baker filled with 20 mL of H_2O_2 and 400 mL of ice. After thoroughly washing with 0.1 M HCl solution and DI water,

the obtained product was redistributed in the water by ultrasonic and then freeze-drying to obtain a fluffy faint yellow powder. Subsequently, the yellow power product was put into quartz tube furnace and heat treated at 900 ℃ for one hour in an inert atmosphere and then subjected to ammonia annealing (30 min) at 900 ℃ to obtain the final product. For comparison, the N-doped CNT (N-CNT, the direct ammonia treatment of pristine CNT) and undoped ultranarrow graphene nanoribbons (D-UGNR, without ammonia injection) were also prepared.

The fabrication of DN-UGNR was illustrated in **Schematic S1**. First, the CNTs was put into the mixture acid solution to remove the impurities. In addition, some defects and holes would be exposed. After adding permanganate into the concentrated acid, the manganate ester would be formed in the dehydrating medium.[1, 2] These defective positions are more prone to be attacked by manganate ester.[1-3] Hence, an opening has been initiated. As the process continues, the opening would be gradually increased. When the nanotubes are fully unzipped, the ultranarrow graphene nanoribbons would be formed.[2, 4] At the same time, owing to the harsh concentrated acid medium and high temperature, these ultranarrow graphene nanoribbons would be severely oxidized and corroded. Thus, the obtained product has abundant oxygen-containing groups (hydroxyl, carbonyl and carboxyl) and defects (e.g., edges, holes, and vacancies),[2, 5] which is labeled as ultranarrow graphene oxide nanoribbons (UGONR). Thirdly, the UGONR was thermally treated at the inert high temperature to remove the surface groups, leading to generate a large number of the defective product (D-UGNR). Subsequently, the D-UGNR was subjected to ammonia treatment at high temperature to obtain the DN-UGNR catalyst.

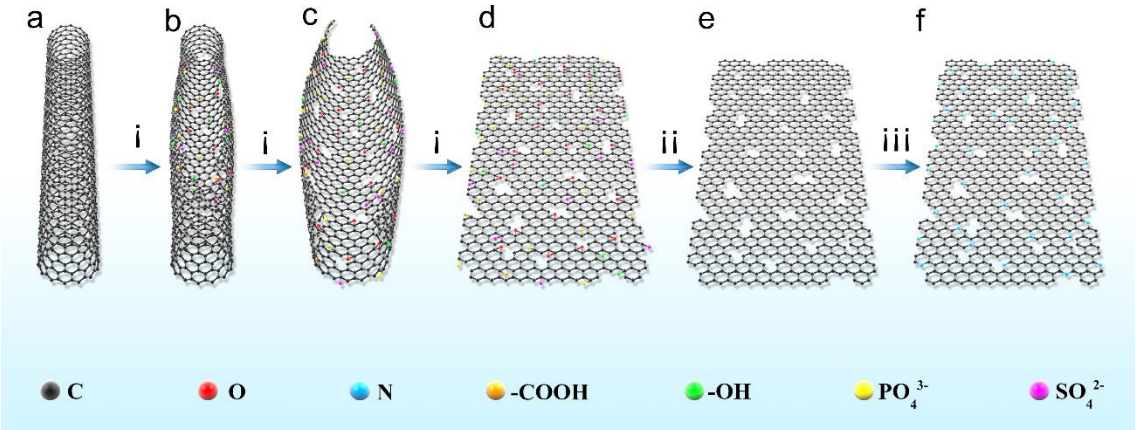

Schematic S1 The formation mechanism of DN-UGNR. a: pristine CNTs, b and c: the partial unzipped CNTs, d: the fully unzipped CNTs, also named as UGONR, e: D-UGNR, f: DN-UGNR. ¡: the mixed concentrated acid (H_2SO_4 and H_3PO_4) and $KMnO_4$, ¡¡: the thermal treatment at 900 ℃ (1 h), ¡¡¡: the ammonia injection at 900 ℃ (30 min).

The preparation of other widths of defective and N-doped GNRs.

DN-GNR(6nm): The raw carbon nanotubes was changed as the diameter of ca. 7 nm, and the length of 10-15 μm. The other conditions are the same as the DN-UGNR.

DN-GNR(12nm): The raw carbon nanotubes were changed as the diameter of 10-12 nm, and the length of 5-15 μm. The "unzipped" reaction time was enlarged to 1.5 hours. The other conditions are the same as the DN-UGNR.

DN-GNR(24nm): The raw carbon nanotubes were changed as the diameter of 10-20 nm, and the length of 5-15 μm. The "unzipped" reaction time was enlarged to 2 hours. The other conditions are the same as the DN-UGNR.

Characterization

The high-resolution transmission electron microscope (TEM, JEM-2100F) was carried out to observe the microstructures of the carbon paper-based materials. The X-ray diffractometer (XRD) patterns were performed using a Rigaku X-ray diffractometer equipped with Cu Ka radiation source. Raman spectra were collected using an argon ion laser operated at a wavelength of 514.5 nm. X-ray photoelectron

spectroscopy (XPS) measurements were determined by ESCALab MKII electron spectrometer with an excitation source of Mg Ka radiation (1253.6 eV). N_2 adsorption and desorption isotherms were conducted on Micromeritics ASAP 2020 analyzer at 77 K. Electron paramagnetic resonance (EPR) spectra were obtained using Bruker EMXnano wave spectrometer at room temperature.

Electrochemical Measurements

All the electrochemical measurements were carried out using an electrochemical workstation (CHI 760E) with a typical three-electrode system at the room temperature. A platinum wire, Ag/AgCl (3 M KCl) and glassy carbon disk (5 mm in diameter) were employed as a counter, reference, and the working electrode, respectively. All potentials in this study were corrected to a reversible hydrogen electrode (RHE). The as-prepared catalysts (5.0 mg) were mixed with ethanol (0.950 mL) and Nafion solution (50 μL, 5 wt %, DuPont) to form a homogeneous ink. For comparison, the commercial noble metal catalysts (Pt/C (Johnson Matthey, 20%) or RuO_2 (Macklin)) was also tested. For noble metal catalysts (Pt/C and RuO_2), the loading is 0.1 mg cm^{-2}. For carbon-based non-noble metal catalyst, the loading is 0.3 mg cm^{-2}. Here, the carbon black (XC-72, 20 wt%) was added into the RuO_2 ink to improve the electron conductivity of catalyst. The electrocatalytic activity was mainly investigated by cyclic voltammetry (CV) and linear sweep voltammetry (LSV) measurements. CV curves were measured in O_2 saturated 0.1 M KOH solution with a scan rate of 20 mV s^{-1}. LSV curves were tested in O_2 saturated 0.1 M KOH solution by using the rotating disk electrode (RDE) technique with a sweep speed of 10 mV s^{-1} and the rotation rate at 1600 rpm. For ORR stability, the difference value of half-wave potential ($E_{1/2}$) was derived from the LSV plots of the catalyst before and after 3000 potential cycles between 0 V to 1.2 V in oxygen-saturated 0.1 M KOH electrolyte. For OER stability, the difference value of the potential at 10 mA cm-2 (E_{10}) was derived from the LSV plots of the catalyst before and after 3000 potential cycles between 1.1 V to 2.0 V in oxygen-saturated 0.1 M KOH.

For the Zn-air battery test, the air cathode was prepared by evenly covering the catalyst ink onto a carbon gas diffusion layer (Teflon-coated carbon fiber paper). The loading is 1 mg cm^{-2} for all the materials. A Zn foil was employed as the anode. Zn-air batteries were assembled by using the above two electrodes in 6 M KOH aqueous electrolyte. Measurements were performed on a home-made electrochemical cell at room temperature.

Density Functional Theory Calculations

All the density functional theory (DFT) calculations were performed by using the Vienna Ab initio Simulation Package (VASP)[6], employing the projected augmented wave (PAW)[7] model. The revised Perdew-Burke-Ernzerhof (RPBE)[8] functional was used to describe the exchange and correlation effect. In all the cases, the cutoff energy was set to be 400 eV. The bulk single-layer graphene was optimized using a 9 × 9 × 1 Monkhorst−Pack[9] k-point mesh, obtaining a lattice constant of graphene 2.47 Å. The periodic surfaces were modeled using 4 × 7 and 3 × 6 supercells for the zigzag and armchair edges, respectively. Of all the surface calculations, a 3 × 1 × 1 mesh was adopted, reciprocally proportional to the surface parameters. When optimizing the surface structures, the edge atoms and the adsorbates were allowed to fully relax until reaching the convergence tolerance; while the other atoms were fixed in their bulk arrangement. The force and energy tolerance was set to be 0.05 eV $Å^{-1}$ and 10^{-5} eV, respectively.

The free energy of OH adsorption on the slabs was defined as

$$\Delta G_{ads} = \Delta E_{ads} + \Delta E_{ZPE} - T\Delta S_{ads}$$

where ΔE_{ads} is the electronic adsorption energy, ΔE_{ZPE} is the zero-point energy difference between adsorbed and gaseous species, and $T\Delta S_{ads}$ is the corresponding entropy difference between these two states.

The binding energy of OH on the slabs was defined as

$$\Delta E_{ads} = E[slab+OH] - E[slab] - E[O] - E[H]$$

where E[slab+OH], E[slab], E[O], and E[H] represent the electronic energy of the

adsorption system, the clean slab, the energy difference between H_2O and gaseous H_2, and half the energy of gaseous H_2, respectively. Under this definition, a lower binding energy indicates a stronger adsorption configuration. More details about this model can be found in the supporting information.

In alkaline conditions, OER could occur in the following four elementary steps:

$OH^- + * \rightarrow *OH + e^-$

$*OH + OH^- \rightarrow *O + H_2O + e^-$

$*O + OH^- \rightarrow *OOH + e^-$

$*OOH + OH^- \rightarrow * + O_2 + H_2O + e^-$

Meanwhile, the ORR is the reverse reaction of OER and contains the following four steps:

$O_2 + H_2O + e^- \rightarrow *OOH + OH^-$

$*OOH + e^- \rightarrow *O + OH^-$

$*O + H_2O + e^- \rightarrow *OH + OH^-$

$*OH + e^- \rightarrow * + OH^-$

where * denotes the active sites on the catalyst surface. Based on the above mechanisms, the free energy of three intermediate states, *OH, *O, and *OOH, is important to identify a given material's activity towards OER and ORR. The computational hydrogen electrode (CHE) model[10] was used to calculate the free energies of the intermediate states, based on which the free energy of an adsorbed species is defined as

$$\Delta G_{ads} = \Delta E_{ads} + \Delta E_{ZPE} - T\Delta S_{ads}$$

where ΔE_{ads} is the electronic adsorption energy, ΔE_{ZPE} is the zero-point energy difference between adsorbed and gaseous species, and $T\Delta S_{ads}$ is the corresponding entropy difference between these two states. The electronic binding energy is referenced as ½ H_2 for each H atom, and (H_2O – H_2) for each O atom, plus the energy of the clean slab. The corrections of zero point energy and entropy of the OER intermediates can be found in **Table S4**.

Equation S1 and **S2**

The Koutecky-Levich (K-L) equation as given below:

$$\frac{1}{J}=\frac{1}{J_L}+\frac{1}{J_K}=\frac{1}{B\omega^{1/2}}+\frac{1}{J_K} \qquad (1)$$

$$B=0.62nFC_0(D_0)^{2/3}\upsilon^{-1/6} \qquad (2)$$

where J denotes the measured current density, J_K is the kinetic current density, J_L is the diffusion-limited current density, ω is the electrode rotation rate, F is the Faraday constant (96485 C mol^{-1}), C_0 is the bulk concentration of O_2, D_0 is the diffusion coefficient of O_2, v is the kinetic viscosity of the electrolyte. In alkaline electrolyte (0.1 M KOH), C_0 is 1.2×10^{-3} mol L^{-1}, D_0 is 1.9 $\times10^{-5}$ cm^2 s^{-1} and v is 1.0×10^{-2} cm^2 s^{-1}.

Equation S3 and **S4**

The rotating ring-disk electrode (RRDE) measurements for the catalyst was also performed with a three-electrode system in O_2-saturated 0.1 M KOH solution at a rotation rate of 1600 rpm with a scan rate of 10 mV s^{-1}, and the potential of the Pt ring was set at V = 1.5 V. The electron transfer numbers were calculated based on the following equations:

$$n=4*J_D/(J_D+J_R/N) \qquad (3)$$

$$H_2O_2\%=200J_R/(N\bullet J_D+J_R) \qquad (4)$$

Represent the disk and ring currents respectively, and N is the current collection efficiency of the Pt ring, which was 0.37 in our system.

Figure captions in Supporting Information

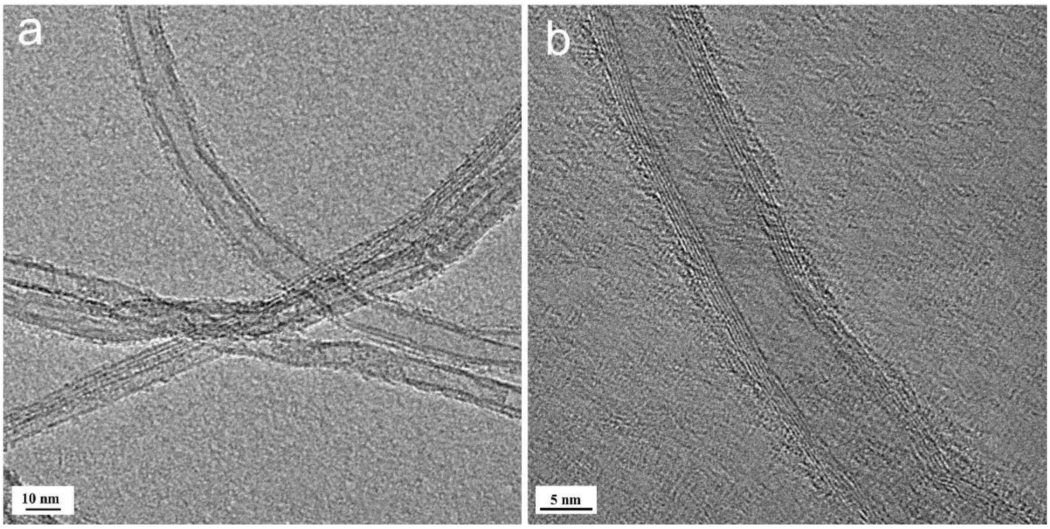

Figure S1 TEM image of pristine CNT.

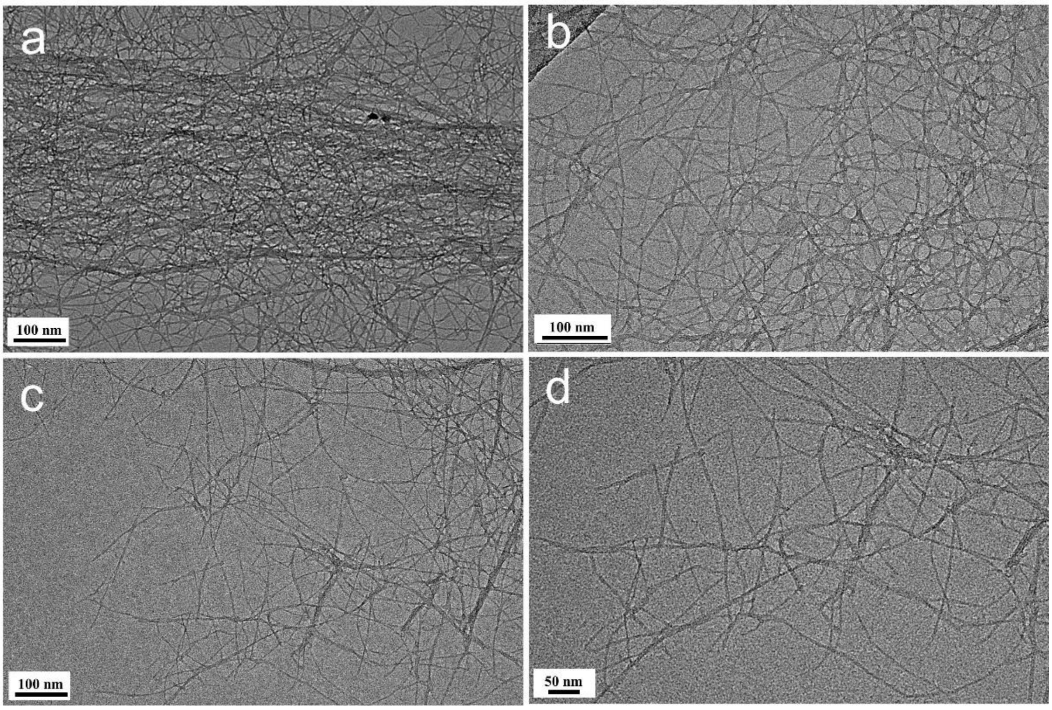

Figure S2 TEM image of DN-UGNR.

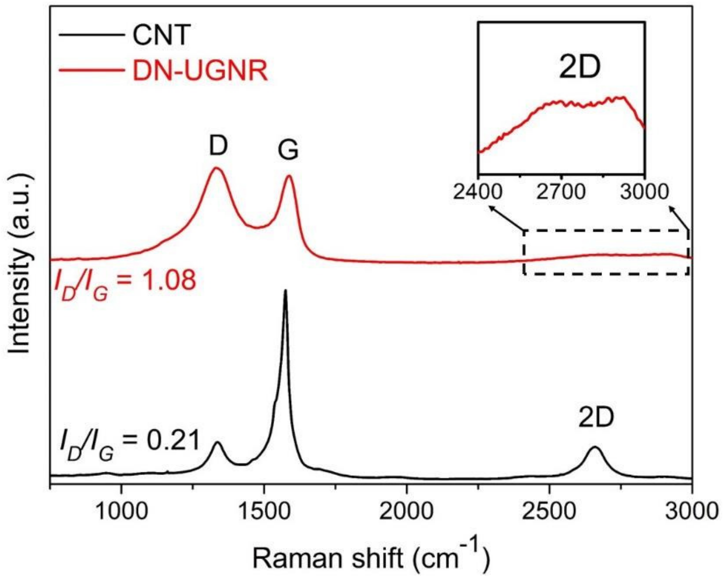

Figure S3 Raman spectra of CNT and DN-UGNR.

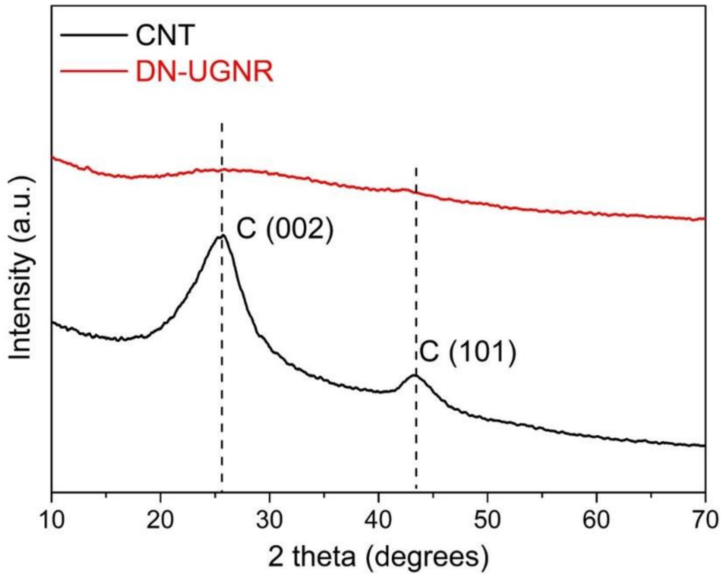

Figure S4 XRD pattern of CNT and DN-UGNR.

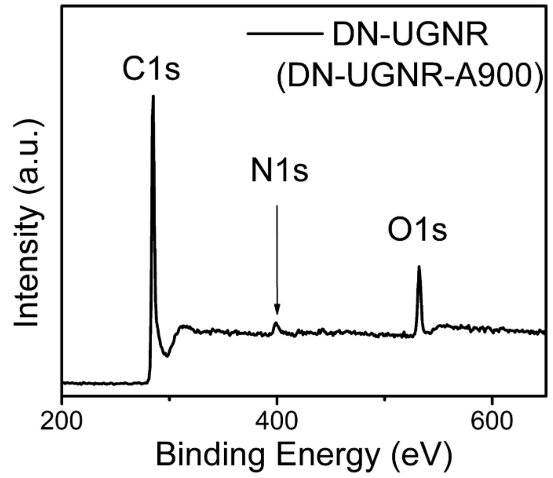

Figure S5 XPS spectrum of DN-UGNR.

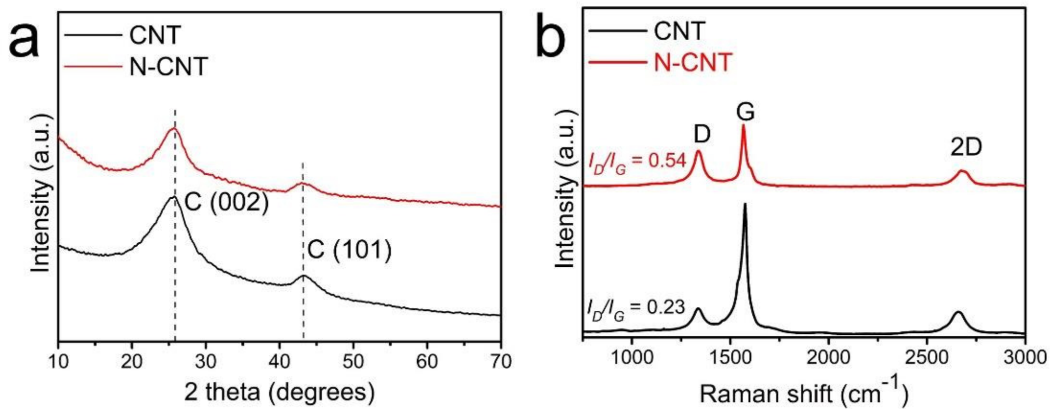

Figure S6 (a) XRD pattern and (b) Raman spectra of CNT and N-CNT.

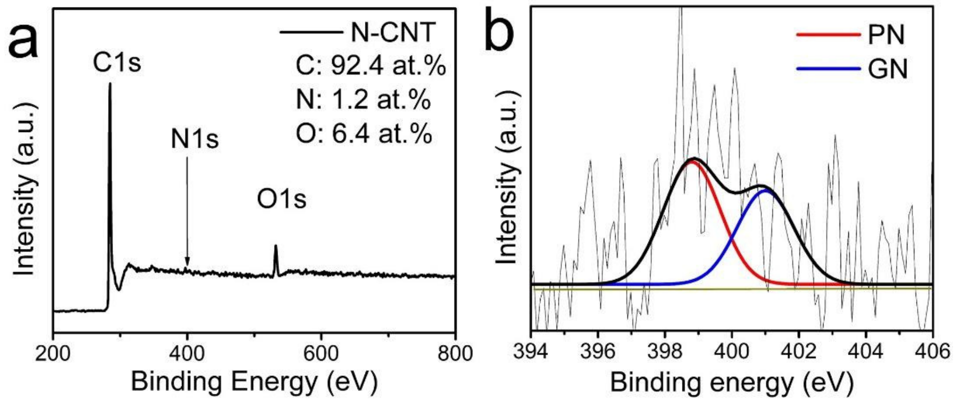

Figure S7 (a) XPS spectrum and (b) the N 1s fitted result of N-CNT.

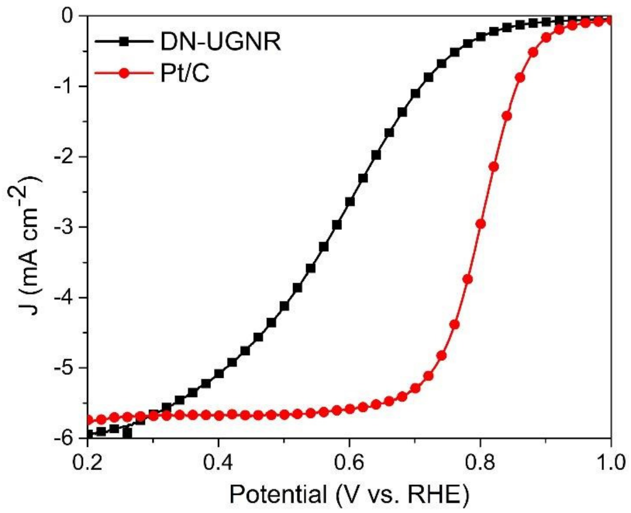

Figure S8 LSV curves for DN-UGNR and Pt/C catalysts at 1600 rpm in O_2-saturated 0.1 M $HClO_4$ solution.

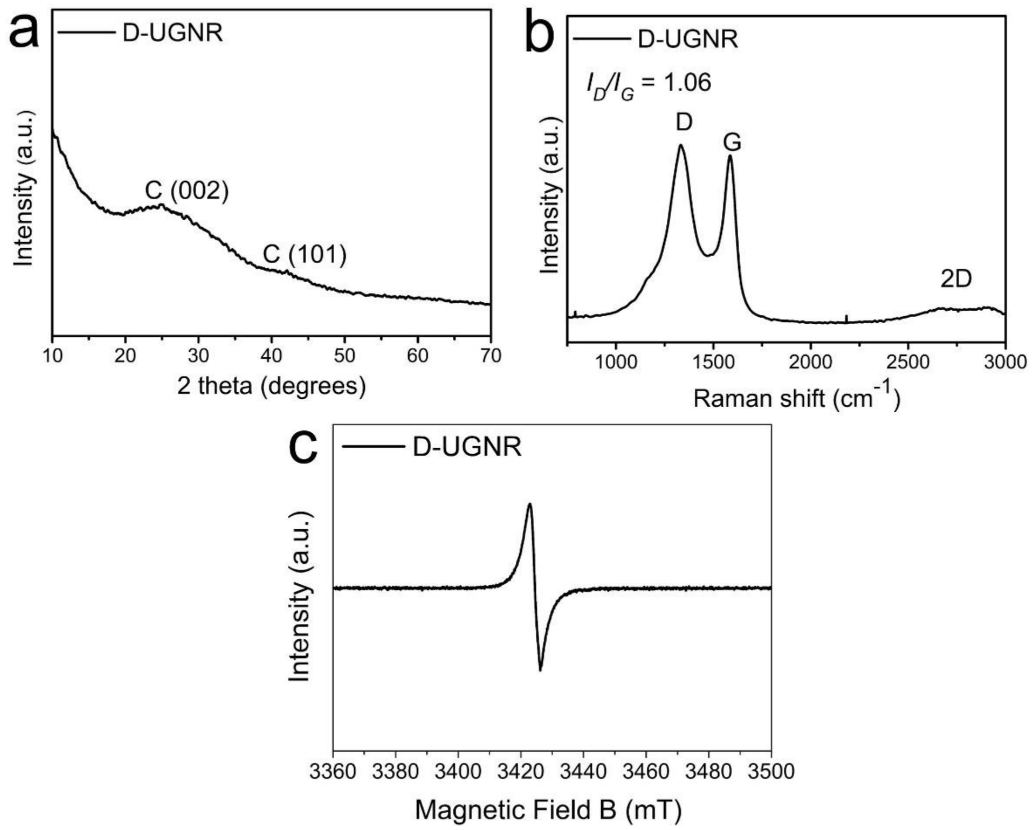

Figure S9 (a) XRD pattern, (b) Raman and (c) EPR spectra of D-UGNR.

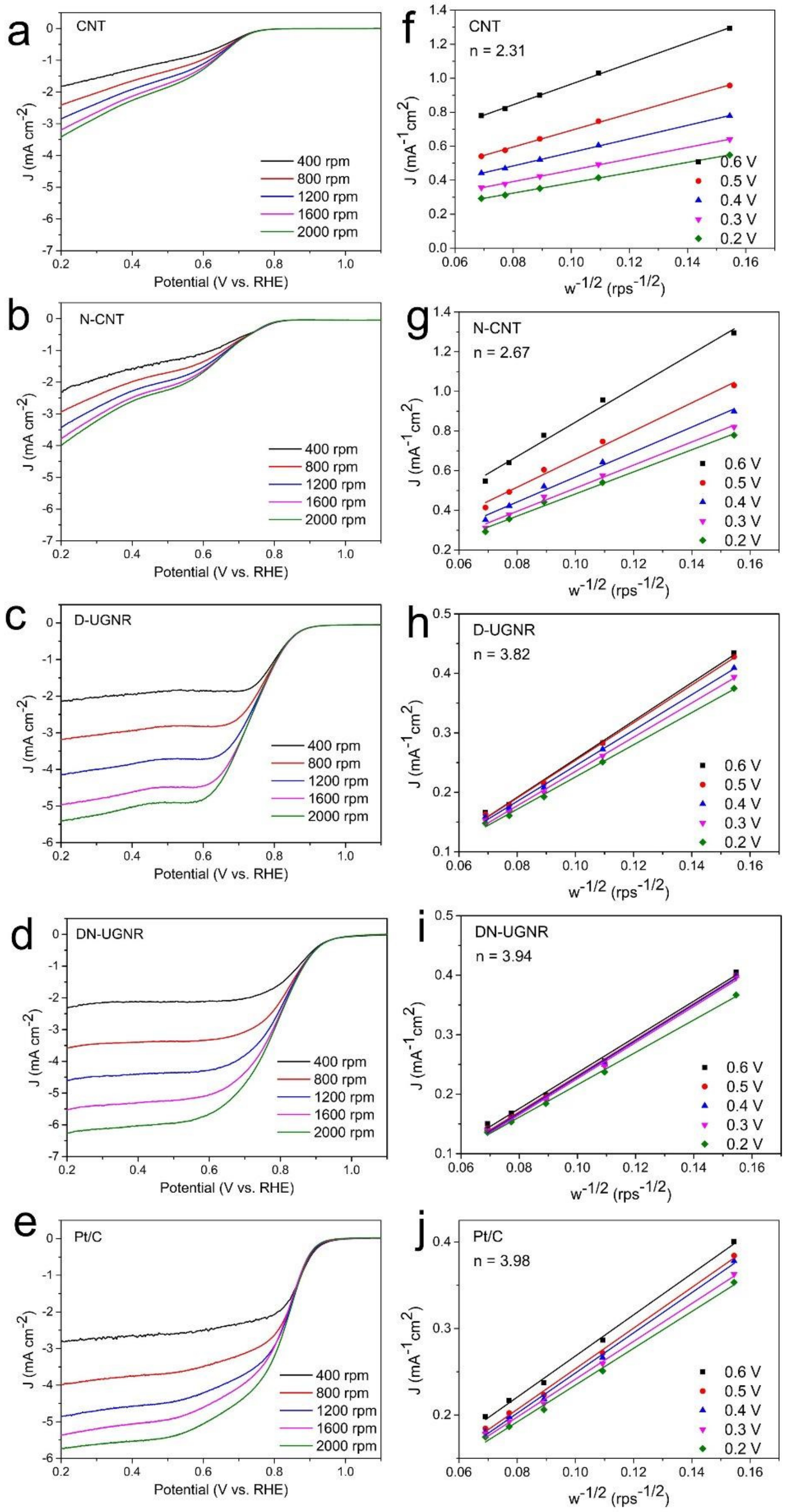

Figure S10 (a-e) LSV curves and (f-j) K-L plots for CNT, N-CNT, D-UGNR, DN-UGNR and Pt/C catalysts.

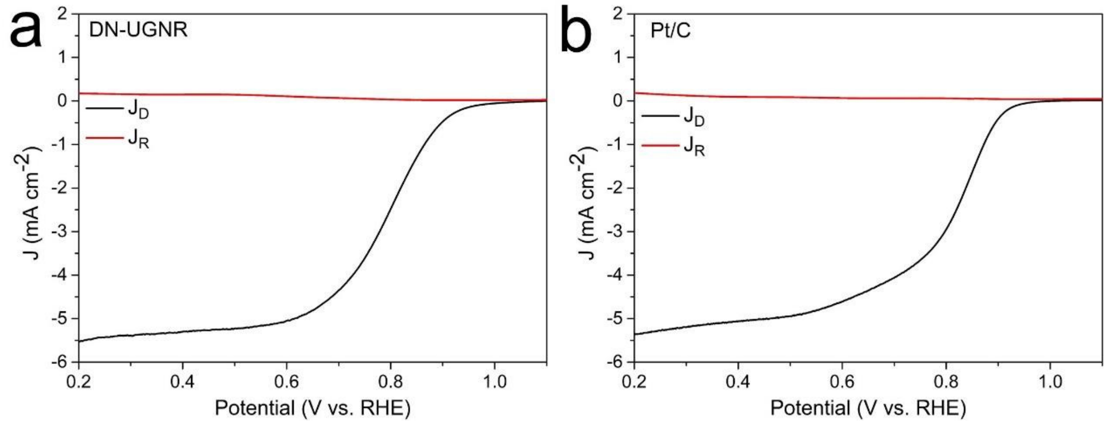

Figure S11 RRDE measurement of (a) DN-UGNR and (b) Pt/C catalysts.in O_2-saturated 0.1 M KOH at 1600 rpm, where J_D is the disk current density, J_R is ring current density.

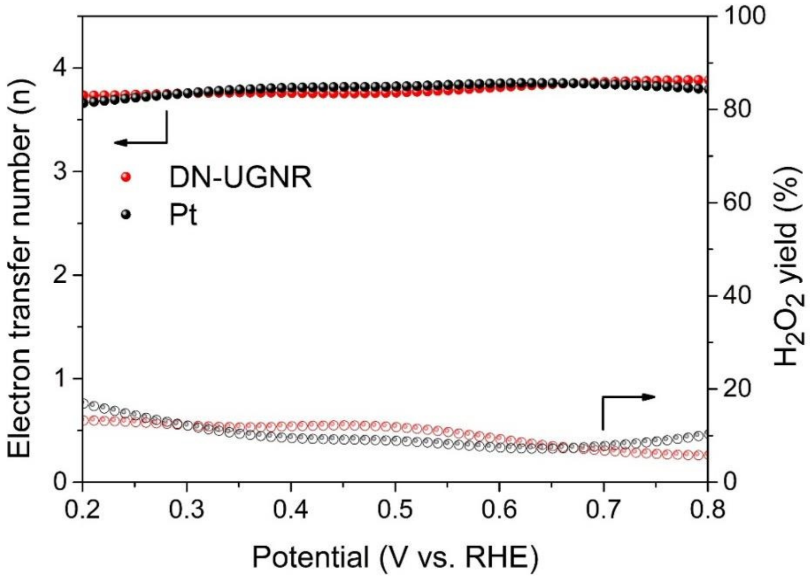

Figure S12 The electron transfer numbers and peroxide yields of DN-UGNR and Pt/C catalysts.

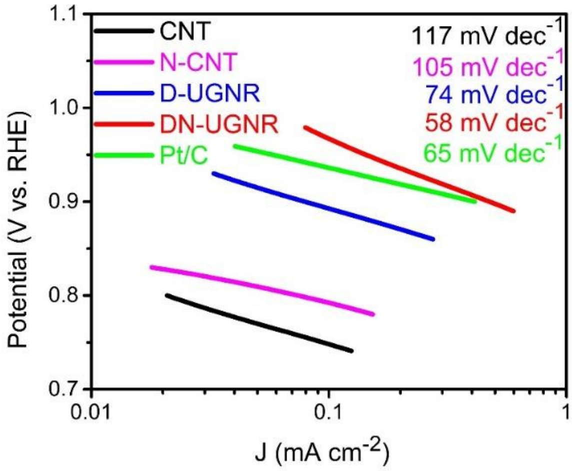

Figure S13 Tafel plots of CNT, N-CNT, D-UGNR, DN-UGNR and Pt/C catalysts.

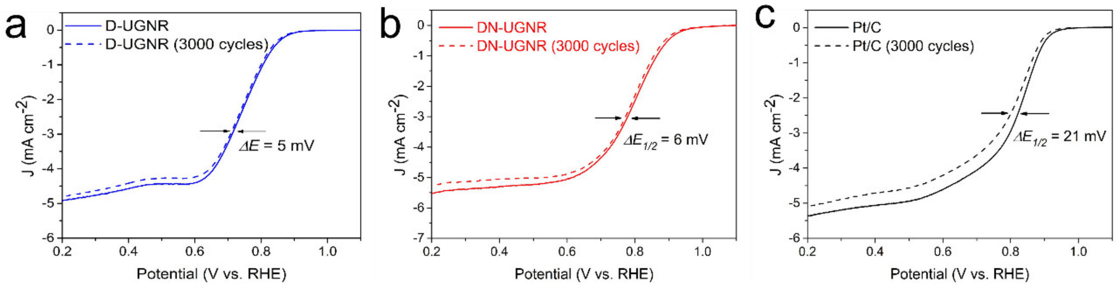

Figure S14 The ORR stability of (a) D-UGNR, (b) ND-UGNR and (c) Pt/C catalysts was symbolized by the decay of half-wave potential ($\Delta E_{1/2}$) before and after cycling test of 3000 cycles.

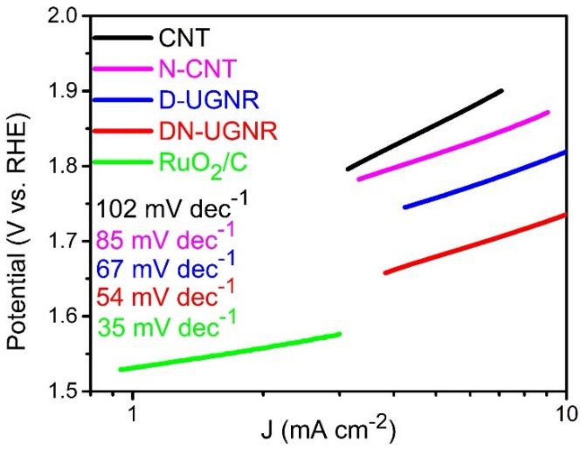

Figure S15 Tafel plots of CNT, N-CNT, D-UGNR, DN-UGNR and RuO2/C catalysts.

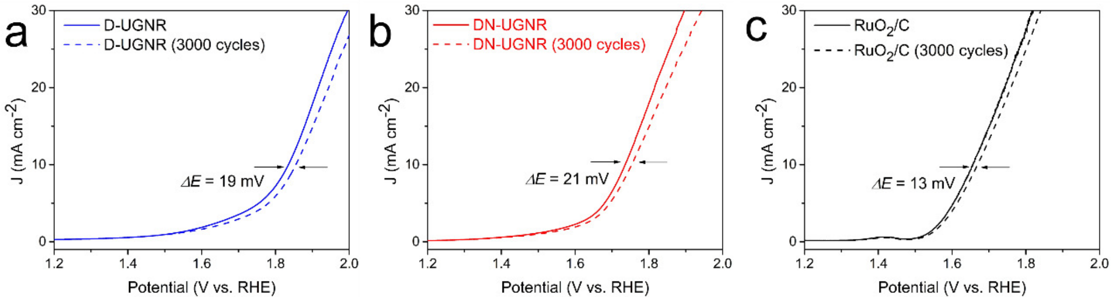

Figure S16 The OER stability of (a) D-UGNR, (b) ND-UGNR and (c) RuO_2/C catalysts was symbolized by the decay of half-wave potential ($\Delta E_{1/2}$) before and after cycling test of 3000 cycles.

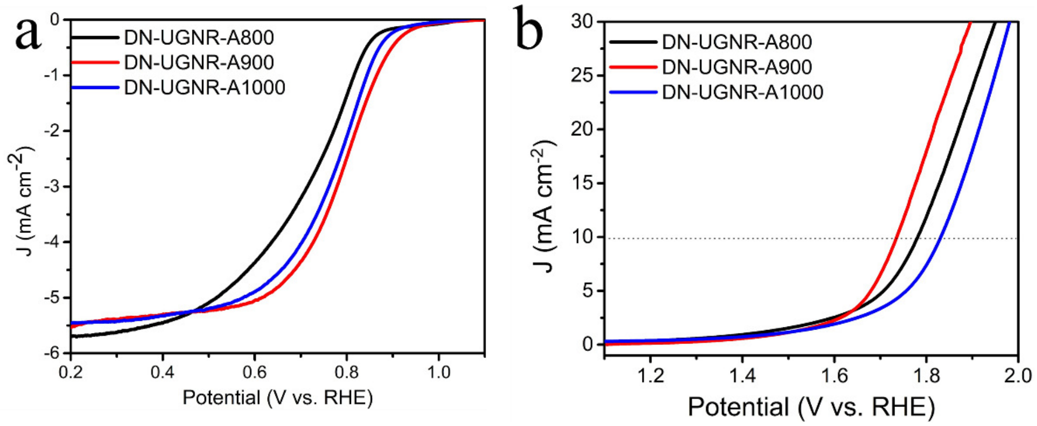

Figure S17 (a) ORR and (b) OER performance of DN-UGNR-A800, DN-UGNR-A900 (also named as DN-UGNR) and DN-UGNR-A1000 catalysts.

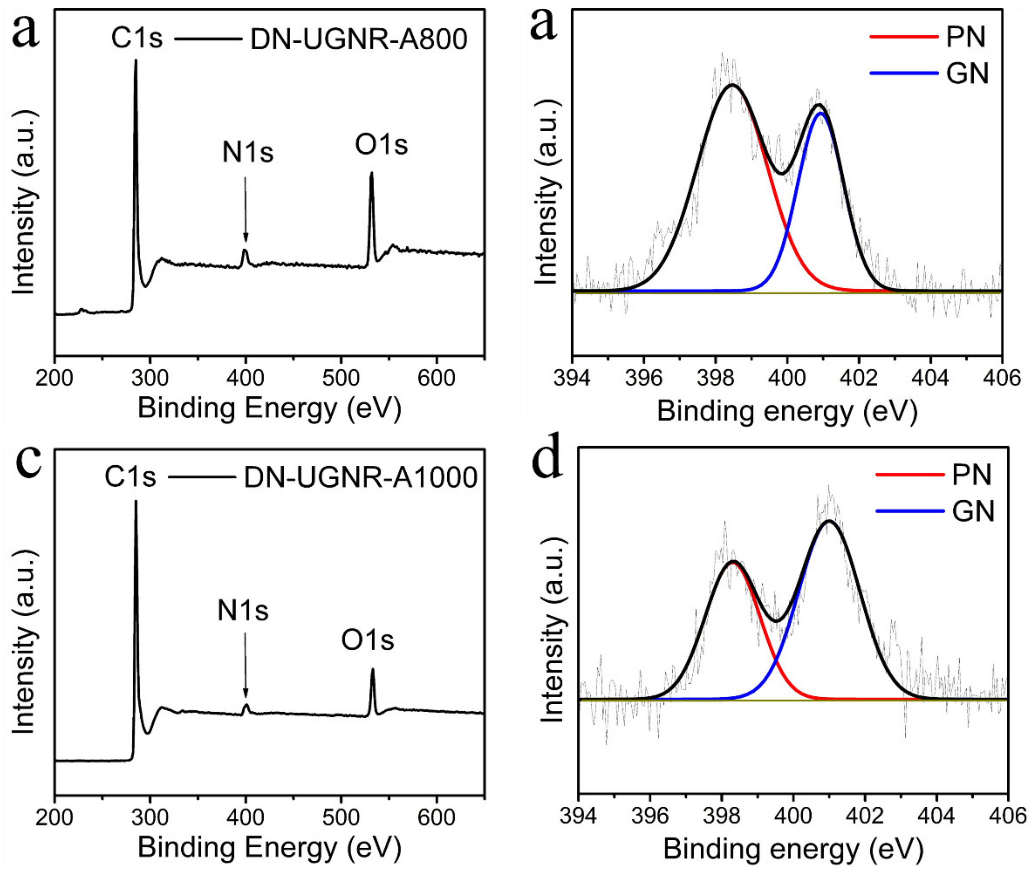

Figure S18 The survey XPS spectra and N 1s fitted results of (a,b) DN-UGNR-A800 and (c,d) DN-UGNR-A1000 catalysts.

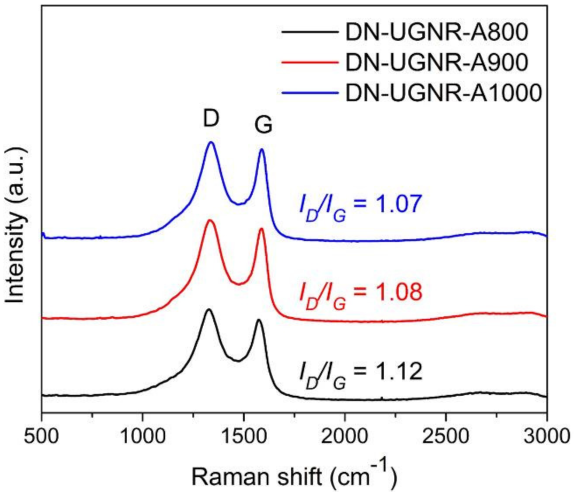

Figure S19 Raman spectra of DN-UGNR-A800, DN-UGNR-A900 (also named as DN-UGNR) and DN-UGNR-A1000 catalysts.

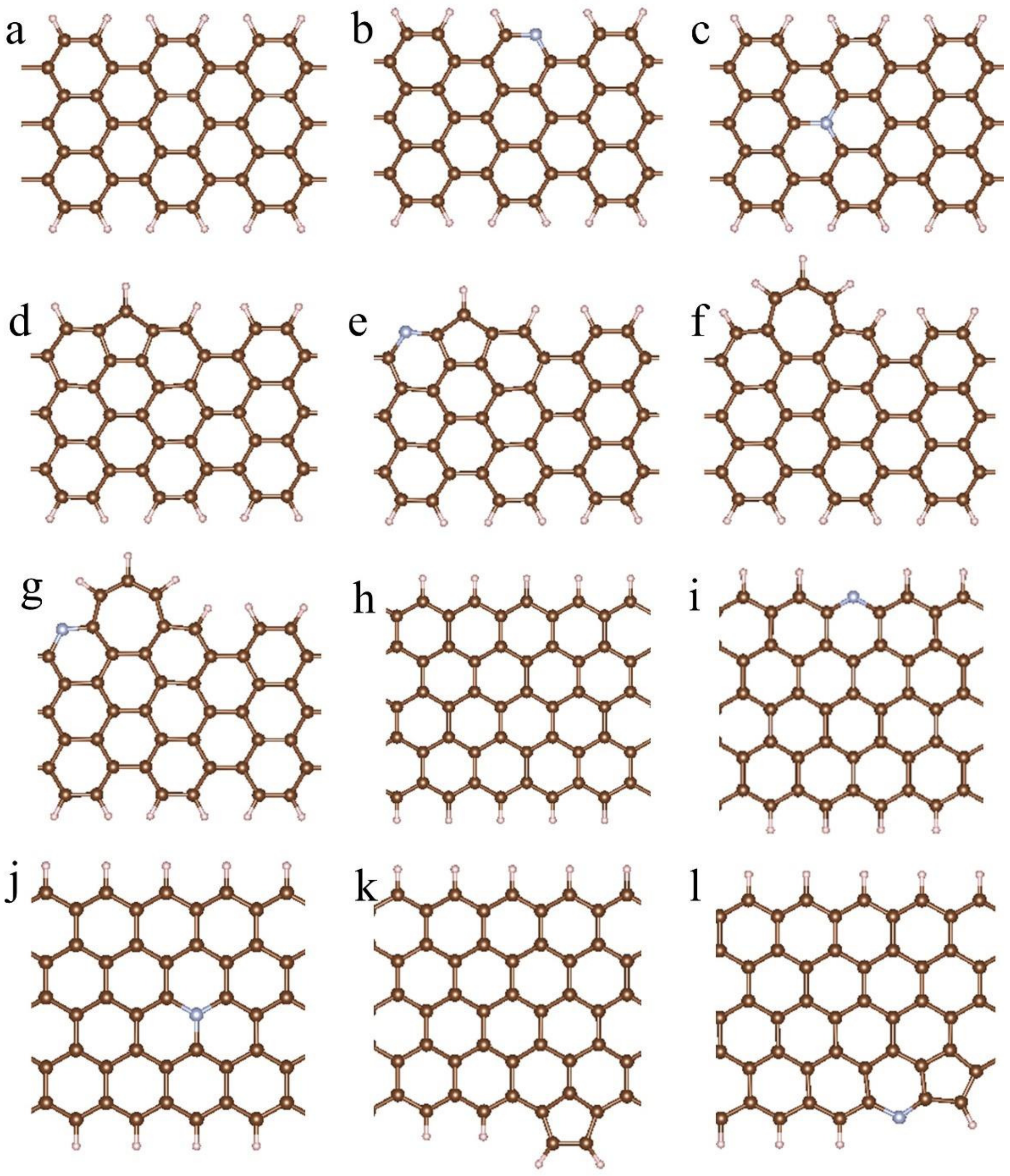

Figure S20 Models for DFT calculations. (a) A: armchair edge, (b) A-PN: pyridinic-N on the armchair edge, (c) A-GN: graphitic-N in the armchair edge, (d) A-C5: pentagon defect on the armchair edge, (e) A-PN+C5: the composite of pyridinic-N and pentagon defect on the armchair edge, (f) A-C7: heptagon defect on the armchair edge, (g) A-PN+C7: the composite of pyridinic-N and heptagon defect on the armchair edge, (h) Z: zigzag edge, (i) Z-PN: pyridinic-N on the zigzag edge, (j) Z-GN: graphitic-N in the zigzag edge, (k) Z-C5: pentagon defect on the zigzag edge, (l) Z-PN+C5: the composite of pyridinic-N and pentagon defect on the zigzag edge.

Table S1 The width range of the graphene nanoribbons.

Reference	Reference number in the text	Width range (nm)
This work	0	1-5
[11]	1	1-10
[12]	2	1.5-50
[13]	3	6-20
[14]	4	20-40
[15]	5	2-50
[16]	6	30-45
[17]	7	20-40
[18]	8	40-60
[19]	9	80-120
[20]	10	100-120
[21]	11	100-500
[22]	12	50-400
[23]	13	50-300
[24]	14	300-500

Table S2 The surface atoms content and the fitted N 1s result of the DN-UGNR-A800, DN-UGNR-A900 and DN-UGNR-A1000.

Samples	Content (at. %)			N species (%)	
	C	N	O	PN	GN
DN-UGNR-A800	80.3	5.5	14.2	64.1	35.9
DN-UGNR-A900	85.3	4.8	9.9	62.7	32.3
DN-UGNR-A1000	89.8	2.9	7.3	40.7	59.3

Table S3 ORR and OER performance of the various electrocatalysts in 0.1 M KOH solution. Where the E_0 and E_{10} are represented the onset potential of ORR and the

overpotential of OER (the geometric current density of 10 mA cm^{-2}), respectively.

Catalyst	E_0 (V)	E_{10} (V)	Reference electrode	Ref.
DN-UGNR	0.957	0.512	RHE	This work
DG	0.91	0.43	RHE	[25]
NT-G	0.99		RHE	[26]
Fe3-NG	0.996		RHE	[27]
NGM-Co	0.84	0.46	RHE	[28]
NDGs-800	0.98		RHE	[29]
P-G	0.912		RHE	[30]
NGM	0.89	0.48	RHE	[31]
P-CC	0.76		RHE	[32]
N-GRW	0.92	0.35	RHE	[33]
BClCNTs-2	0.94		RHE	[34]
DN-CP@G	0.92	0.558	RHE	[5]
NKCNPs-900		0.49	RHE	[35]
N/C		0.42	RHE	[36]
FeCo-Nx-CN	0.954	0.51	RHE	[37]
GO-PANi31-FP	0.85	0.52	RHE	[38]

Table S4 The correction of zero point energy and entropy of the adsorbed and gaseous species.

	ZPE(eV)	TS(eV)
*OOH	0.35	0
*O	0.05	0
*OH	0.31	0.01

H_2O	0.56	0.67
H_2	0.27	0.41

References

[1] D. V. Kosynkin, A. L. Higginbotham, A. Sinitskii, J. R. Lomeda, A. Dimiev, B. K. Price, J. M. Tour, Nature 2009, 458, 872.

[2] A. L. Higginbotham, D. V. Kosynkin, A. Sinitskii, Z. Sun, J. M. Tour, ACS Nano 2010, 4, 2059.

[3] R. Cruz-Silva, A. Morelos-Gomez, S. Vega-Diaz, F. Tristan-Lopez, A. L. Elias, N. Perea-Lopez, H. Muramatsu, T. Hayashi, K. Fujisawa, Y. A. Kim, M. Endo, M. Terrones, ACS Nano 2013, 7, 2192.

[4] Q. Peng, Y. Li, X. He, X. Gui, Y. Shang, C. Wang, C. Wang, W. Zhao, S. Du, E. Shi, P. Li, D. Wu, A. Cao, Adv. Mater. 2014, 26, 3241.

[5] C. Hang, J. Zhang, J. Zhu, W. Li, Z. Kou, Y. Huang, Adv. Energy Mater. 2018, 1703539.

[6] G. Kresse, J. Furthmüller, Physical Review B 1996, 54, 11169; G. Kresse, J. Hafner, Physical Review B 1994, 49, 14251.

[7] P. E. Blöchl, Physical Review B 1994, 50, 17953.

[8] J. P. Perdew, K. Burke, M. Ernzerhof, Physical Review Letters 1996, 77, 3865; Y. Zhang, W. Yang, Physical Review Letters 1998, 80, 890.

[9] H. J. Monkhorst, J. D. Pack, Physical review B 1976, 13, 5188.

[10]J. K. Nørskov, J. Rossmeisl, A. Logadottir, L. Lindqvist, J. R. Kitchin, T. Bligaard, H. Jonsson, The Journal of Physical Chemistry B 2004, 108, 17886.

[11]L. Jiao, L. Zhang, X. Wang, G. Diankov, H. Dai, Nature 2009 GNR 1nm, 458, 877.

[12]X. Li, X. Wang, L. Zhang, S. Lee, H. Dai, Science 2008 GNR 1.5-50nm, 319, 1229.

[13]J. Lim, U. N. Maiti, N. Y. Kim, R. Narayan, W. J. Lee, D. S. Choi, Y. Oh, J. M. Lee, G. Y. Lee, S. H. Kang, H. Kim, Y. H. Kim, S. O. Kim, Nat. Commun. 2016, 7,

10364.

[14] H. B. Yang, J. Miao, S. F. Hung, J. Chen, H. B. Tao, X. Wang, L. Zhang, R. Chen, J. Gao, H. M. Chen, L. Dai, B. Liu, Sci. Adv. 2016, 2, e1501122.

[15] T. H. Vo, M. Shekhirev, D. A. Kunkel, M. D. Morton, E. Berglund, L. Kong, P. M. Wilson, P. A. Dowben, A. Enders, A. Sinitskii, Nat. Commun. 2014, 5, 3189.

[16] M. Liu, Y. Song, S. He, W. W. Tjiu, J. Pan, Y. Y. Xia, T. Liu, ACS Appl. Mater. Interfaces 2014, 6, 4214.

[17] L. Chen, R. Du, J. Zhu, Y. Mao, C. Xue, N. Zhang, Y. Hou, J. Zhang, T. Yi, Small 2015, 11, 1423.

[18] Q. Peng, Y. Li, X. He, X. Gui, Y. Shang, C. Wang, C. Wang, W. Zhao, S. Du, E. Shi, P. Li, D. Wu, A. Cao, Adv. Mater. 2014, 26, 3241.

[19] A. Zehtab Yazdi, E. P. L. Roberts, U. Sundararaj, Carbon 2016, 100, 99.

[20] Y. Gong, H. Fei, X. Zou, W. Zhou, S. Yang, G. Ye, Z. Liu, Z. Peng, J. Lou, R. Vajtai, B. I. Yakobson, J. M. Tour, P. M. Ajayan, Chemistry of Materials 2015, 27, 1181.

[21] J. Annett, G. L. Cross, Nature 2016, 535, 271.

[22] D. V. Kosynkin, A. L. Higginbotham, A. Sinitskii, J. R. Lomeda, A. Dimiev, B. K. Price, J. M. Tour, Nature 2009 GNR principle, 458, 872.

[23] A. L. Higginbotham, D. V. Kosynkin, A. Sinitskii, Z. Sun, J. M. Tour, ACS Nano 2010 GONR preparation 4, 2059.

[24] R. Cruz-Silva, A. Morelos-Gomez, S. Vega-Diaz, F. Tristan-Lopez, A. L. Elias, N. Perea-Lopez, H. Muramatsu, T. Hayashi, K. Fujisawa, Y. A. Kim, M. Endo, M. Terrones, ACS Nano 2013, 7, 2192.

[25] Y. Jia, L. Zhang, A. Du, G. Gao, J. Chen, X. Yan, C. L. Brown, X. Yao, Adv. Mater. 2016, 28, 9532.

[26] Y. Li, W. Zhou, H. Wang, L. Xie, Y. Liang, F. Wei, J. C. Idrobo, S. J. Pennycook, H. Dai, Nat. Nanotechnol. 2012, 7, 394.

[27] X. Y. Cui, S. B. Yang, X. X. Yan, J. G. Leng, S. Shuang, P. M. Ajayan, Z. J. Zhang, Adv. Funct. Mater. 2016, 26, 5708.

[28] C. Tang, B. Wang, H. F. Wang, Q. Zhang, Adv. Mater. 2017 ECSA, 29.

[29] Q. Wang, Y. Ji, Y. Lei, Y. Wang, Y. Wang, Y. Li, S. Wang, ACS Energy Letters 2018, 1183.

[30] L. Tao, Q. Wang, S. Dou, Z. Ma, J. Huo, S. Wang, L. Dai, Chem. Commun. 2016, 52, 2764.

[31] C. Tang, H. F. Wang, X. Chen, B. Q. Li, T. Z. Hou, B. Zhang, Q. Zhang, M. M. Titirici, F. Wei, Adv. Mater. 2016, 28, 6845.

[32] Z. Liu, Z. Zhao, Y. Wang, S. Dou, D. Yan, D. Liu, Z. Xia, S. Wang, Adv. Mater. 2017, 29, 1606207.

[33] H. B. Yang, J. Miao, S. F. Hung, J. Chen, H. B. Tao, X. Wang, L. Zhang, R. Chen, J. Gao, H. M. Chen, L. Dai, B. Liu, Sci. Adv. 2016, 2, e1501122.

[34] Z. Kou, B. Guo, D. He, J. Zhang, S. Mu, ACS Energy Letters 2017, 184.

[35] Q. Wang, Y. Lei, Y. Zhu, H. Wang, J. Feng, G. Ma, Y. Wang, Y. Li, B. Nan, Q. Feng, Z. Lu, H. Yu, ACS Appl. Mater. Interfaces 2018.

[36] Y. Zhao, R. Nakamura, K. Kamiya, S. Nakanishi, K. Hashimoto, Nat. Commun. 2013, 4, 2390.

[37] S. Li, C. Cheng, X. Zhao, J. Schmidt, A. Thomas, Angewandte Chemie 2018, 57, 1856.

[38] J. Zhang, L. Dai, Angewandte Chemie 2016, 55, 13296.
